# The impact of task measurements on sequential dependence: a comparison between temporal reproduction and discrimination tasks

**DOI:** 10.1007/s00426-024-02023-x

**Published:** 2024-08-27

**Authors:** Si Cheng, Siyi Chen, Xuefeng Yang, Zhuanghua Shi

**Affiliations:** 1 General and Experimental Psychology, Department of Psychology, LMU Munich, 80802 Munich, Germany; 2 Graduate School of Neural & Behavioural Science, International Max Planck Research School, Tübingen, Germany

## Abstract

Decisions about a current stimulus are influenced by previously encountered stimuli, leading to sequential bias. However, the specific processing levels at which serial dependence emerges remain unclear. Despite considerable evidence pointing to contributions from perceptual and post-perceptual processes, as well as response carryover effects impacting subsequent judgments, research into how different task measurements affect sequential dependencies is limited. To address this gap, the present study investigated the role of task type in shaping sequential effects in time perception, employing a random-dot kinematogram (RDK) in a post-cue paradigm. Participants had to remember both the duration and the direction of the RDK movement and perform the task based on a post-cue, which was equally likely to be direction or duration. To delineate the task type, we employed the temporal bisection task in Experiment 1 and the duration reproduction task in Experiment 2. Both experiments revealed a significant sequential bias: durations were perceived as longer following longer previous durations, and vice versa. Intriguingly, the sequential effect was enhanced in the reproduction task following the same reproduction task (Experiment 2), but did not show significant variation by the task type in the bisection task (Experiment 1). Moreover, comparable response carryover effects were observed across two experiments. We argue that the differential impacts of task types on sequential dependence lies in the involvement of memory reactivation process in the decision stage, while the post-decision response carryover effect may reflect the assimilation by subjective, rather than objective, durations, potentially linking to the sticky pacemaker rate and/or decisional inertia.

**Supplementary Information:**

The online version contains supplementary material available at 10.1007/s00426-024-02023-x.

## Introduction

Our decisions about a current stimulus are influenced by previously encountered events, resulting in a reliable yet biased estimation known as “serial dependence” or “sequential dependence” (Cicchini et al., 2014, 2023; Fischer & Whitney, 2014; Glasauer & Shi, 2022; Pascucci et al., 2023). Extensive research has demonstrated the widespread phenomenon of serial dependence using visual features (Bae & Luck, 2020; Barbosa & Compte, 2020; Fischer & Whitney, 2014). Such history dependence and trial-to-trial influences have also been observed in time perception (Glasauer & Shi, 2022; Shi et al., 2013; Togoli et al., 2021; Wehrman et al., 2023). For instance, subjective duration can be biased by recent history (Burr et al., 2009; Jazayeri & Shadlen, 2010; Nakajima et al., 1992), leading to the central tendency effect – underestimating long durations and overestimating short ones (Glasauer & Shi, 2021; Hollingworth, 1910). Unlike the central tendency effect, sequential dependence specifically refers to the influence of recent trials on the current trial (Glasauer & Shi, 2022; Wehrman et al., 2023; Wiener et al., 2014). Although serial dependence is generally acknowledged, the processing levels at which it emerges remain unclear. Additionally, research on how different task measurements affect sequential dependencies is limited.

There are two main perspectives: the perceptual account and the post-perceptual account. The perceptual account suggests that sequential dependence promotes perceptual stability and temporal continuity by integrating past and current information to filter out abrupt noises, functioning mainly as a perceptual rather than decision-making mechanism (Cicchini et al., 2017; Fornaciai & Park, 2018a; Glasauer & Shi, 2022; Liberman et al., 2016). For instance, research has identified behavioral or neural signatures of serial dependence that occur independently of any response requirement (Czoschke et al., 2019; Fornaciai et al., 2023; Fornaciai & Park, 2018a; Pascucci et al., 2024). Generally, these studies involve experiments where participants focus on a single type of stimulus and often just report one feature, while not always needing to respond (Czoschke et al., 2019; Fischer & Whitney, 2014). However, focusing on a single feature might blur the lines between perceiving and reporting it, and the frequent need to report a target feature might prime participants toward preparing responses even when none are needed, potentially impacting the logic of the interpretation.

Conversely, an alternative perspective attributes sequential effects to decision-related post-perceptual factors (Bae & Luck, 2020; Ceylan et al., 2021; Ceylan & Pascucci, 2023; Fritsche & de Lange, 2019; Pascucci et al., 2019; Ranieri et al., 2022; Suárez-Pinilla et al., 2018). This perspective gains support from studies investigating how task-relevant responses might influence serial dependence when responses involve multiple target feature dimensions (Bae & Luck, 2020; Fischer et al., 2020; Houborg et al., 2023; Suárez-Pinilla et al., 2018; Togoli et al., 2021). This approach reflects real-world scenarios where individuals typically encounter and remember various features of objects simultaneously. For example, as you wait at a crossroad for the traffic light to turn green, you monitor not just its color but also how long it remains on each signal. In such contexts, judging color and judging duration impact consequent estimations of each differently. A recent study explored this by having participants engage with two features: duration and motion direction, and perform either duration or motion adjustment tasks according to cues presented either before or after the target stimuli were shown (Cheng et al., 2024). Their findings indicated that sequential dependence in timing tasks was mainly evident when consecutive tasks involved the same duration tasks but diminished when the task types varied, even when participants attentively encoded both features in a post-cue setup.

The varying impacts of task types on serial dependence may also depend on the specific tasks used to assess sequential biases. For example, Pascucci et al. (2023) reviewed recent studies on serial dependence and revealed that the effect depends on whether the task is a reproduction or a forced-choice task. In reproduction tasks, participants replicate the perceived attribute of a stimulus, whereas forced-choice tasks require participants to make binary decisions, judging if the stimulus differs from a standard reference in predefined ways (e.g., shorter vs. longer, larger vs. smaller, etc.). The effects of task-relevant responses on serial dependence are not consistent between these two types of tasks. For example, studies using reproduction tasks have found serial dependence to be influenced by prior choices and post-perceptual decisions (Bae & Luck, 2020; Cheng et al., 2023). In contrast, other studies using forced-choice tasks show that serial dependence can manifest even without explicit responses (Fornaciai & Park, 2018a).

This variation in findings could be attributed to how each task type interacts with working memory. Reproduction tasks may demand ongoing comparisons between the stimulus being reproduced and a memorized one, whereas forced-choice tasks typically require a single, direct comparison of sensory input against a reference, minimizing the need for post-stimulus retention. Additionally, the decision strategies employed in these tasks could differ significantly (Gokaydin et al., 2011; Lages & Treisman, 1998; Sumner & Sumner, 2020); reproduction tasks require a thorough encoding of the entire stimulus before it can be accurately reproduced, whereas forced-choice tasks may allow for quicker decision-making based on a decision threshold without full stimulus encoding. For example, in short/long timing tasks, participants need not encode the entire duration of the stimulus that lasts longer than a midpoint of the short and long references, given that the “long” decision can already be made. Therefore, the choice of “task” is a crucial factor for understanding the role of task-relevant response in sequential effect. Yet, the role of task types in sequential dependence in time perception hasn’t been investigated.

While task types may potentially impact sequential dependence and decision-making, post-decision responses may impact the upcoming judgments directly. Recent studies have shown that responses from previous trials could significantly influence outcomes in subsequent trials (Li et al., 2023; Wehrman et al., 2020, 2023). For example, the prior judgment of a duration as “Long” (or “Short”) is likely carried over to the next trial, regardless of preceding durations (Wehrman et al., 2020, 2023; Wiener et al., 2014). This indicates that subjective durations, rather than physical durations, also impact subsequent decision-making (Wehrman et al., 2023). This response carryover may also reflect the observer’s inclination to maintain a self-consistent interpretation of the world (Luu & Stocker, 2018), operating under the assumption that the state of the world tends to remain constant (similar argument is also in Glasauer & Shi, 2022), which leads to the observed post-decision biases. Given the carryover of post-decision responses is primarily determined by the response state rather than task types or memory processes, the sequential response carryover might be independent of task types, presenting a complex issue that remains unresolved.

On this ground, we designed two experiments to investigate how different task types - specifically, the duration reproduction and bisection tasks, randomly intermixed with non-timing direction tasks - affect sequential effect and decision carryover in duration judgments. Specifically, we employed the random-dot kinematogram (RDK), incorporating two features: motion direction and timing, in a post-cue setup. Participants had to remember its duration and direction during the encoding phase, reporting one according to post cues. In Experiment 1, we randomly intermixed temporal bisection trials with non-timing direction-adjustment trials, while in Experiment 2, we intermixed duration reproduction trials with the direction-adjustment trials. We hypothesized that the extent to which working memory is involved plays a critical role in sequential dependence (Cheng et al., 2023; Pascucci et al., 2023). Unlike the forced-choice bisection task (categorizing durations as either “Short” vs. “Long”), the duration reproduction requires reactivation of the encoded duration from working memory (Bae & Luck, 2019; Barbosa & Compte, 2020). Consequently, we expect an enhanced sequential effect if consecutive tasks involve the same duration reproduction, compared to when tasks alternate between timing and non-timing tasks. In contrast, the temporal bisection task requires only maintaining a decisional state (either “Short” or “Long”) that is likely made during the encoding stage, without further resorting to the memory reactivation process. Of note, decisions can be made even before the complete presentation in some long-duration trials during the encoding phase. Therefore, we anticipate that the sequential dependence, if any, may be less affected by task switching or repetition. On the response level, we presume that the reproduced duration in the reproduction task implicitly represents subjective durations. By categorizing these subjective responses into “short” or “long” categories, we expect to observe comparable decision carryover effects across two task types, assuming that decision carryover effects are primarily influenced by response states rather than memory processing.

## Experiment 1

### Method

#### Participant

Twenty-six volunteers participated in Experiment 1 (14 females and 12 males, ranging in age from 18 to 26 years, with a mean of 20.8 years and a standard deviation of 2.17 years). All participants were right-handed, with normal or corrected-to-normal color vision. We excluded two participants for their large response variability (see the section “Data Analysis”) and reported the results from the remaining 24 participants. We chose the sample size by referencing prior studies (Bae & Luck, 2020; Fischer & Whitney, 2014), which often identify significant effects (Cohen’s d > 0.753). Participants signed the informed consent form before the experiment commenced and received compensation at a rate of 9 Euros/hour. The study was approved by the ethics committees of the Psychology Department at LMU Munich.

#### Stimuli and procedure

We used PsychoPy (Peirce et al., 2019) to manage stimuli presentation and to collect data. Participants were seated approximately 60 cm from the screen in a soundproof, dimly lit cabin. The stimuli were presented on a 24-inch DELL monitor (refresh rate 60 Hz) against a light grey background (39.3 cd/m^2^).

**Fig. 1:**
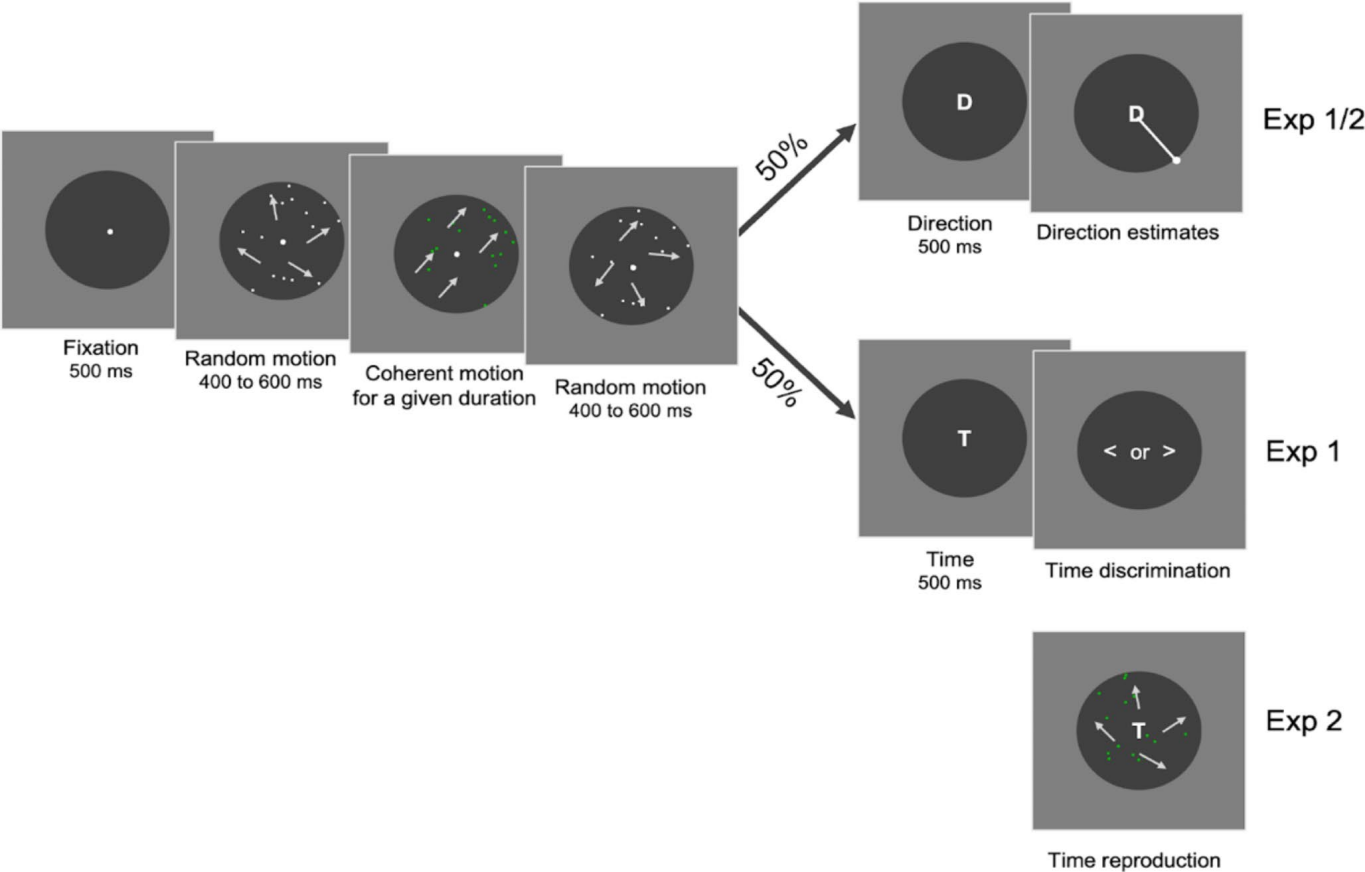
Schematic illustration of the experimental procedure. A trial started with a fixation dot, followed by a white random dot kinematogram. After 400 ms to 600 ms, the dots turned green and moved together in one direction for a given duration; then the display shifted back to the white random dot kinematogram. Next, a cue appeared for half a second, either the letter ‘D’ for the direction task or the letter ‘T’ for the timing task. For the direction task, participants adjusted a line pointer with arrow keys and confirmed their report by pressing the spacebar. Experiments 1 and 2 differed in the timing task. In Experiment 1, it was a discrimination task, with a prompt display (“< or >”), while in Experiment 2, the letter ‘T’ stayed on till the completion of the reproduction task

As outlined in Fig. 1, each trial began with a fixation dot for half a second (0.5° in diameter with a brightness of 85.7 cd/m^2^), which cued the start of the trial and drew participants’ attention. Next came the encoding phase, wherein a random dot kinematogram (RDK) featuring 15 white dots (each dot diameter of 0.4°; the luminance of 85.7 cd/m^2^) against a dark disc (17.8°, 16.5 cd/m^2^) appeared at the center of the screen. Initially, the dots within the RDK moved randomly for 400 to 600 ms, without any pattern (at a speed of 1 °/s and a coherence level of 0%). Subsequently, these dots turned green (45.8 cd/m^2^) and began moving together (at 100% coherence) at a speed of 6°/s in a predetermined direction (randomly selected from 11.25° to 348.75°, in steps of 22.5°) for a randomly chosen length of [0.4, 0.6, 0.8, 1.2, 1.4, 1.6] s. When a dot exited the dark disc boundary, another dot appeared randomly inside to maintain a constant count of fifteen. These green, coherently moving dots served as the target, which participants were asked to memorize regarding their movement direction and duration. After this, the dots returned to their initial random motion for another 400 to 600 ms. The alternating white dot displays served as visual masks to present any residual visual effects from the previous trial.

Following the encoding phase, a post cue - either the letter ‘D’ (0.8° × 1.0°, 85.7 cd/m^2^) for the direction task or ‘T’ for the time task - appeared at the center of the display for half a second, prompting participants to report either the direction or duration. Participants could respond at their own pace. For the duration discrimination task, a display showing the left and the right arrows (“< or >”) prompted participants to assess if the duration of the coherent motion was shorter or longer than one second. They made this two-alternative forced choice (2AFC) judgment by pressing the left arrow for “shorter than one second” or the right arrow for “longer than one second”.

In the direction task, a line segment started from the center with an overlaid ‘D’, pointing to a random direction. Participants rotated this line to match the observed motion’s direction using the left (counterclockwise) and right (clockwise) arrow keys. A continuous readjustment updated the pointer’s direction, and they finalized their choice by pressing the spacebar. If their estimated direction deviated by more than 60°, a warning message “Direction deviated a lot!” would flash on-screen for half a second. The next trial began after a one-second intertrial interval.

To prepare participants for the main experiment, a practice session with 24 practice trials exposed them to a standard one-second stimulus, represented by yellow dots moving horizontally (at a speed of 6 °/s; coherence of 100%). Following a 500 ms blank interval, a comparison stimulus with a duration randomly chosen of [0.4, 0.6, 0.8, 1.2, 1.4, 1.6] s was presented. The comparison stimulus was the same RDK display used in the main experiment. Participants had to judge which one was longer. After the response, they received feedback on their accuracy. The formal experiment consisted of 480 trials, randomly shuffled, and split evenly between duration and direction tasks. The inter-trial transitional probability (from trial *n-1* to trial *n* ) between the duration and direction trials ensured an equal probability of all inter-trial combinations. Participants could take a short break after each block of 30 trials.

#### Data analysis

In our study, we primarily examined the influence of previous trials on duration judgments within timing tasks. We included the analysis and results for the direction tasks in the supplementary materials for readers interested in exploring this aspect further. For the timing tasks, we categorized trials based on the duration (less than or more than 1 s) and type (Time or Direction) of the previous trial, creating four categories: “Short/Direction”, “Long/Direction”, “Short/Time”, and “Long/Time”. We further classified consecutive Time-Time trials according to the preceding timing trials’ responses as “Short Response” or “Long Response.” Excluding the first trial of each block, we analyzed responses using a psychometric function, a cumulative Gaussian function, including an initial 5% lapse rate for attention errors (Wichmann & Hill, 2001). We then determined each participant’s Points of Subjective Equality (PSE) to identify biases in duration perception and computed the just-noticeable difference (JND) and Weber fraction (WF = JND/PSE) for precision. Two participants with a WF greater than one were excluded for further analysis. Lastly, we used repeated measures ANOVAs and two-sided *t*-tests to determine the significance of our findings.

### Results and discussion

First, we examined whether the difficulty of the two kinds of preceding task (time vs. direction) affected the time discrimination performance in the current trial (Cicchini et al., 2018), and calculated the just-noticeable difference (JND) for Time and Direction conditions, and it didn’t show any significant difference between the two conditions (JND with standard errors for Time: 0.123 ± 0.007, and Direction: 0.129 ± 0.008, *t*_*(23)*_ = 0.589, *p* = .562, *d* = 0.155), suggesting that the task difficulties for time discrimination following Time and Direction conditions were comparable.

**Fig. 2:**
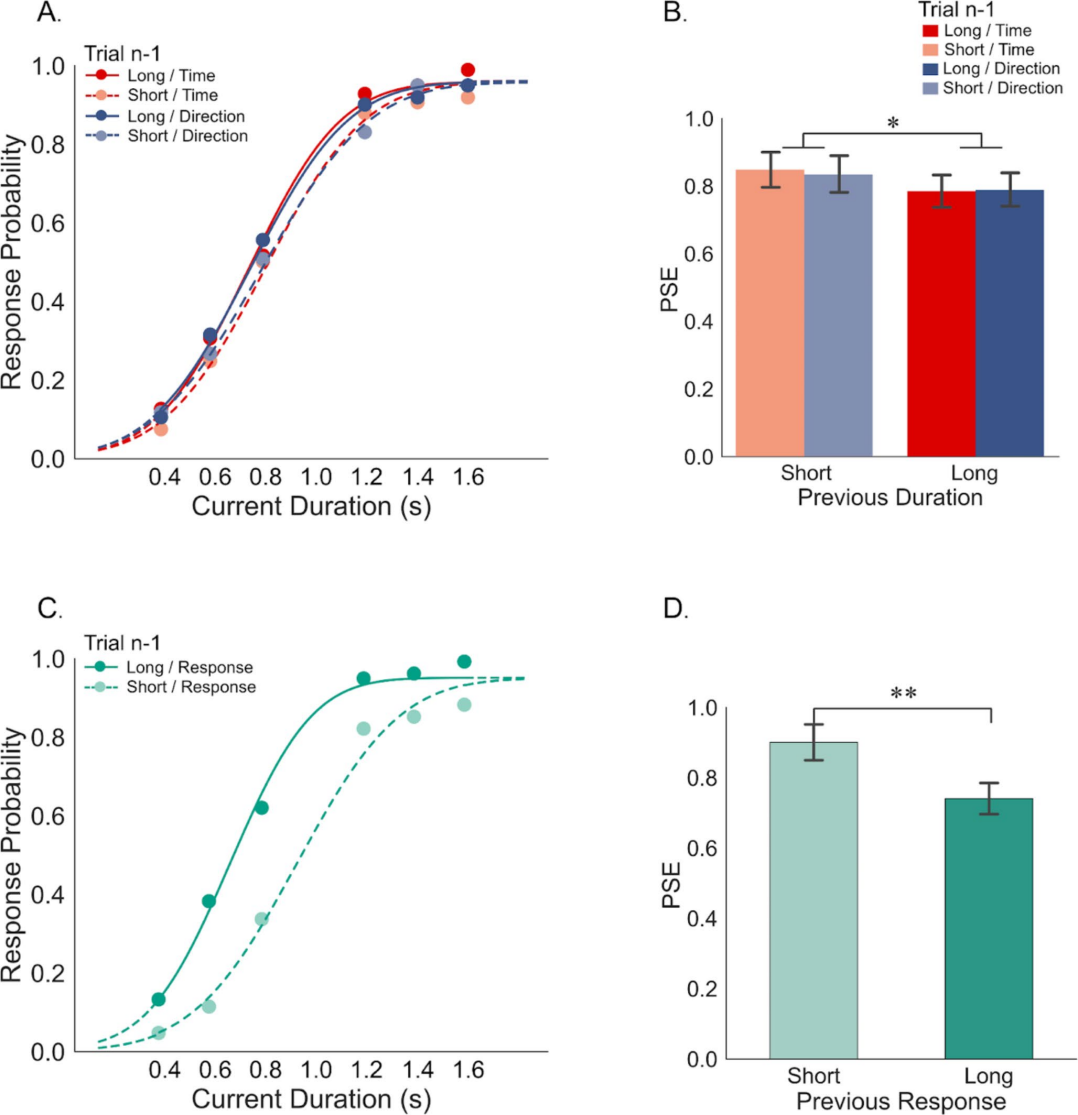
Results of Experiment 1. (**A**). Response probabilities of “Longer than 1 second” on the comparison duration (0.4, 0.6, 0.8, 1.2, 1.4, and 1.6 s) separately for previous time discrimination and direction adjustment tasks when the prior duration was either short (including 0.4, 0.6, and 0.8 s) or long (including 1.2, 1.4, and 1.6 s). The lines show the best-fitting psychometric function. (**B**). Points of subjective equality (PSE) values were plotted for previous time discrimination and direction adjustment tasks when the previous duration was short or long. (**C**). Response probabilities of “Longer than 1 second” on the comparison duration when participants made “Short Response” or “Long Response” in the previous time discrimination trials. The lines show the best-fitting psychometric function. (**D**). Corresponding PSE values for prior “Short Response” and “Long Response.” Error bars represent ± SEM. **p < .01, *p < .05

Then, trials were categorized into four groups based on prior task (Time or Direction) and duration (Short or Long), as shown in Fig. 2A’s psychometric curves. A distinct difference was visible between curves for the preceding “Short” vs. “Long” conditions while preceding “Time” and “Direction” tasks had similar curves. PSEs (with standard errors) were 770 ± 48, 833 ± 51, 775 ± 49, and 820 ± 54 ms for Time/Long, Time/Short, Direction/Long, and Direction/Short, respectively (Fig. 2B). A two-way repeated measures ANOVA revealed a significant effect of Prior Duration, *F*_*(1,23)*_ = 6.083, *p* = .022, p2​​ = 0.012, but not of Prior Task (*F*_*(1,23)*_ = 0.045, *p* = .833, p2 < 0.001) or their interaction (*F*_*(1,23)*_ = 0.138, *p* = .714, p2​​ < 0.001). These findings indicate that prior duration impacts current duration judgment, with shorter prior durations leading to shorter perceived current durations and vice versa, indicating an assimilation bias. The type of prior task (time or direction), however, had little effect.

Further analysis of the impact of preceding responses revealed a decisional carry-over effect. Figure 2C and D show psychometric curves according to prior responses, with a leftward shift for “Long” prior responses. The PSE was significantly lower after “Long” responses (741 ± 44 ms) than “Short” (901 ± 51 ms), *t*_*(23)*_ = 3.795, *p* = .001, *d* = 0.684, indicating a tendency to judge current durations as longer following the “Long” report.

These findings indicate that duration judgments are influenced by both previous durations and decisions, manifesting as both an assimilation effect and a decisional carry-over effect. Importantly, the type of preceding task (Time or Duration) did not significantly impact these biases, suggesting that temporal bisection task, involving binary decision (either “Short” or “Long”), was likely done already in the encoding phase (when the basis of the subsequent judgment isn’t yet known), without needing further involvement of memory reactivation in the reporting stage. In contrast, duration reproduction requires full presentation of the duration and reactivation of the encoded one from working memory during the reproduction stage. This raises the question of whether these findings from Experiment 1 are applicable to a reproduction task. Therefore, Experiment 2 employed a time reproduction task, asking participants to replicate the duration of a given stimulus.

## Experiment 2

### Method

#### Participant

Twenty-four participants were recruited in Experiment 2 (13 females; age 18–27, mean ± SD: 20.75 ± 2.45 years), all right-handed, with normal or corrected-to-normal vision and color vision. Before the experiment, participants provided written informed consent and received 9 Euros/hour compensation.

#### Stimuli and procedure

Experiment 2 closely followed the design of Experiment 1, with the following changes for the timing task. This time, participants had to reproduce the duration of the target stimuli, randomly selected from 0.6, 0.8, 1.0, 1.2, 1.4, 1.6, and 1.8 s (see Fig. 1). After the post-cue display, participants initiated the task at their own pace by pressing and holding the down arrow key, releasing it when they felt the elapsed duration matched the target duration. Immediately after pressing the down arrow key, a display showing static green random dots (15 dots, each dot diameter of 0.4°; the luminance of 45.8 cd/m^2^) turned into a random motion display (velocity of 6 °/s) to minimize inter-trial bias. The key holding duration was recorded as the reproduced duration. If their reproduction error exceeded 30%, they received feedback: “Too short” for relative errors below − 30% and “Too long” for errors above 30%. The procedure for the direction adjustment task remained the same as in Experiment 1.

#### Data analysis

Response errors in duration reproduction trials were calculated as the difference between the reproduced and actual durations. We excluded the first trial of each block and filtered out trials where errors exceeded three standard deviations from the participant’s mean error, accounting for accidental presses or attention lapses. These outliers constituted only 0.39% of trials. The remaining trials were categorized into two conditions based on the prior task (Time or Direction).

Previous research has demonstrated that subjective timing is susceptible to contextual factors, such as the “central tendency effect”, leading to underestimating long durations and overestimating short durations (Burr et al., 2009; Jazayeri & Shadlen, 2010; Nakajima et al., 1992), and the sequential effect, where reproductions are influenced by preceding durations (Dyjas et al., 2012; Glasauer & Shi, 2022). We modeled these effects using multiple linear regressions, with current (T_n_) and previous (T_n-1_) durations as predictors:

1 $${\rm{Error_{n} = a*T_{n} + b*T_{n - 1} + c}}{\rm{.}}$$

The model’s slope (*a*) for the current duration indicates the central tendency effect. Following the convention adopted in the literature (Cicchini et al., 2012; Jazayeri & Shadlen, 2010; Shi et al., 2013), we used the positive value (|*a|*) as the central tendency index, with 0 indicating no central tendency. The slope (*b*) for the previous duration reflects the sequential bias (Cicchini et al., 2014; Glasauer & Shi, 2022), and a positive slope indicates that the current estimation is attracted towards the previous duration, denoted as the “assimilation”, while a negative slope indicates that the current time estimation is repelled from the previous duration. Lastly, we used repeated measures ANOVAs and two-sided *t*-tests to determine the significance of our findings. The statistical significance of the central tendency effect and the sequential effect was assessed individually using two-sided *t*-tests against a null hypothesis of zero effect, and paired *t*-tests were run for within-subject between-condition comparisons.

Furthermore, we categorized reproduced durations as “Longer” or “Shorter” than the middle duration 1.2 s (omitting 1.2 s) and analyzed sequential effects based on prior stimuli and responses, such that we can compare sequential effects between Experiments 1 and 2. Additionally, to visualize the variability of the sequential effect between experiments, we computed a sequential effect index as the difference in PSEs between groups with prior short and prior long durations for each prior task condition. To assess the decisional carry-over effect between experiments, we calculated a decisional carry-over effect index as the difference in PSEs between prior short and prior long reports separately for each experiment. We used repeated measures ANOVAs and two-sided *t*-tests to determine the significance of our findings.

### Results and discussion

The overall mean response error (with SE) for the duration reproduction trials was significantly positive (97 ± 25 ms, *t*_(23)_ = 3.911, *p* = .001, *d* = 0.798), indicating a general overestimation. The mean reproduction error for the prior Time task was 113 ± 24 ms, significantly larger than the mean error for the prior Direction task (78 ± 27 ms), *t*_(23)_ = 3.393, *p* = .003, *d* = 0.278. To examine the variability of duration reproduction for two kinds of preceding task (time vs. direction), we calculated the standard deviation (STD) of reproduction between Time and Direction conditions, and it didn’t show any significant difference between the two conditions (STD with standard errors for Time: 0.289 ± 0.016, and Direction: 0.289 ± 0.016, *t*_*(23)*_ = 0.027, *p* = .979, *d* = 0.003).

**Fig. 3:**
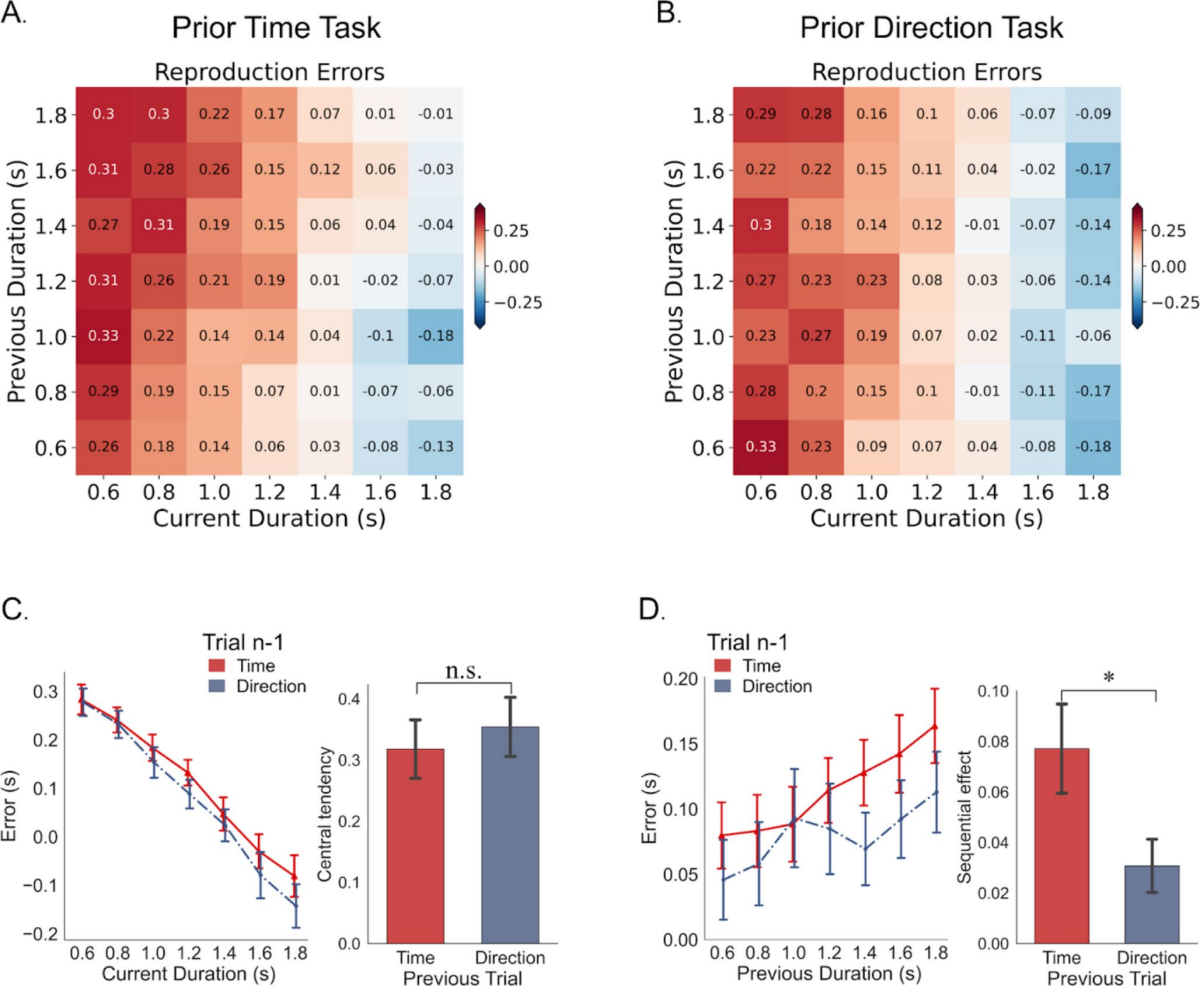
Results of Experiment 2. (**A**) Mean reproduction errors for all participants as a function of current (horizontal axis) and previous (vertical axis) durations for trials preceded by time reproduction task. (**B**) Mean reproduction errors for all participants as a function of current and previous durations for trials preceded by direction adjustment task. The reproduction error decreases as the current duration increases (cells get bluer from left to right, showing central tendency effect), but also becomes more positive as the previous duration increases (cells get redder from bottom to top, indicating sequential dependence effect). (**C**) Central tendency effect. Left panel: mean reproduction errors were plotted on the current sample duration; right panel: index of central tendency effect, plotted separately for trials preceded by time reproduction and direction adjustment tasks. (**D**) Sequential dependence. Left panel: mean reproduction errors were plotted on the previous duration; right panel: index of sequential effect, plotted separately for trials preceded by time reproduction and direction adjustment tasks. Error bars represent ± SEM. *p < .05. n.s. denotes non-significant

Our results showed that both the preceding Time and Direction conditions exhibited central tendency biases and serial dependence effects. As shown in Fig. 3A and B, the average reproduction error decreases as the current duration increases, indicating that participants tend to overestimate short durations and underestimate long durations. Additionally, reproduction errors increased with longer prior durations, indicating an assimilation effect. To illustrate this bias in more detail, take a current trial where the duration is 1.2 s (middle row of Fig. 3A), and it was preceded by a trial with a duration of 1.6 s. In this case, the biased representation of duration takes the value of 1.35 s in the preceding Time condition (the value of 1.31 s in the preceding Direction condition, see Fig. 3B), representing an attractive bias towards the previous trial where the current duration being processed and perceived as being more similar to the previous duration than it actually is. This assimilation bias occurs for both preceding Time and Direction conditions but at different levels. To compare the statistical difference, we quantified the central tendency effect and serial dependence effect using the mean slope |*a|* and *b* from linear regressions (Eq. 1).

*Central tendency effect.* The mean central tendency index (|*a|*) was 0.318 ± 0.048 (*t*_(23)_ = 6.654, *p* < .001, *d* = 1.358) for the Time condition and 0.354 ± 0.048 (*t*_(23)_ = 7.329, *p* < .001, *d* = 1.496) for the Direction condition. They were comparable (*t*_(23)_ = 1.503, *p* = .147, *d* = 0.154), as depicted by the trends in Fig. 3C. This suggests that the task relevance did not influence the central tendency effect. The lack of difference can be attributed to the same distribution and range of durations tested in both tasks, resulting in a stable prior representation of durations across conditions. This finding aligns with previous research that mixing durations leads to generalized prior representation across different conditions (Roach et al., 2017).

*Serial dependence effect*. Figure 3D depicts the assimilation effect of current durations towards prior durations. We quantified this effect using the mean slope *b* from linear regressions, resulting in slopes of 0.077 for prior Time and 0.031 for prior Direction tasks, as shown in Fig. 3D (right panel). Both slopes were significantly greater than zero (Time: *t*_(23)_ = 4.370, *p* < .001, *d* = 0.892; Direction: *t*_(23)_ = 2.921, *p* = .008, *d* = 0.596), confirming a sequential effect in both conditions. Interestingly, the sequential effect was significantly larger in the prior Time relative to the Direction condition (*t*_*(23)*_ = 2.368, *p* = .027, *d* = 0.652). To rule out statistical artifacts (Cicchini et al., 2014), we also analyzed reproduction errors against durations in future trials, which showed no significance (*ps* > 0.460). These findings provide clear evidence that, at least in the case of the time reproduction task, task-relevant response in the preceding trials enhanced the sequential effect.

**Fig. 4:**
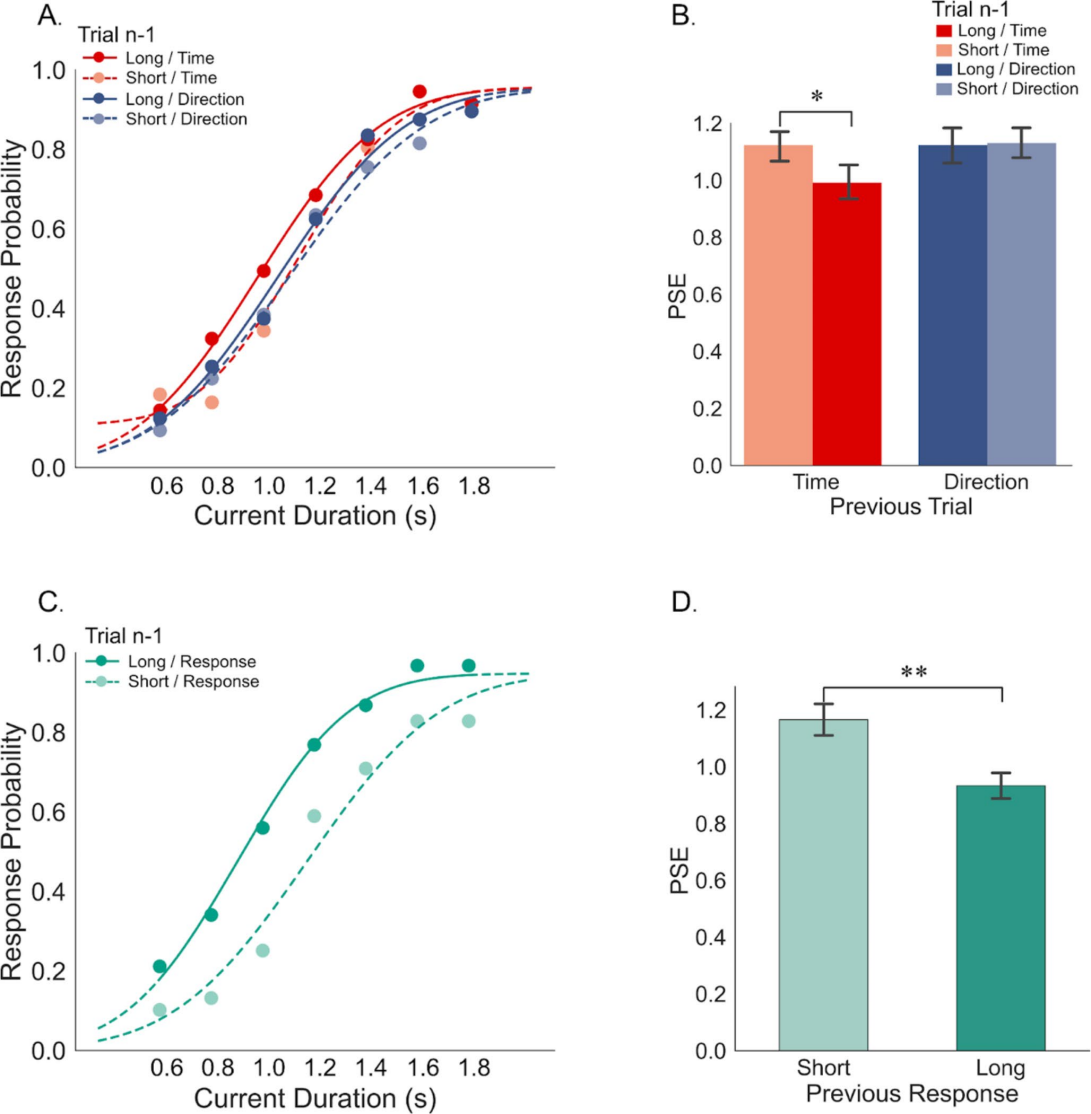
Psychometric function plots of Experiment 2. (**A**) Response probabilities of “Longer than 1.2 second” on the comparison duration (0.6, 0.8, 1.0, 1.2, 1.4, 1.6, and 1.8 s) separately for previous time reproduction and direction adjustment tasks when the prior duration was either short (including 0.6, 0.8, and 1.0 s) or long (including 1.4, 1.6, and 1.8 s). The lines show the best-fitting psychometric function. (**B**) Points of subjective equality (PSE) values were plotted for previous time reproduction and direction adjustment tasks when the previous duration was short or long. (**C**) Response probabilities of “Longer than 1.2 second” on the comparison duration when participants made “Short Response” or “Long Response” in the previous time reproduction trials. The lines show the best-fitting psychometric function. (**D**) Corresponding PSE values for prior “Short Response” or “Long Response”. Error bars represent ± SEM. **p < .01, *p < .05

To compare the findings between Experiments 1 and 2, we categorized reproduced duration as “Short” or “Long” relative to the middle duration 1.2 s. Figure 4A presents psychometric curves that reveal an assimilation bias toward previous durations only in the prior Time condition. In the prior Time condition, the PSE for prior long and short intervals were 987 ± 59 ms and 1111 ± 52 ms, respectively. In the prior Direction condition, these values were 1124 ± 52 ms and 1114 ± 61 ms, respectively (Fig. 4B). A two-way repeated measures ANOVA revealed a main effect of the previous Duration, *F*_*(1,23)*_ = 5.407, *p* = .029, p2 = 0.011, and a main effect of the prior Task, *F*_*(1,23)*_ = 6.150, *p* = .021, p2 = 0.017, and a significant interaction effect between these factors (*F*_*(1,23)*_ = 5.479, *p* = .028, p2 = 0.015). Further analysis revealed a significant assimilation effect in the task-relevant (Time) condition (*t*_(23)_ = 3.465, *p* = .004, *BF*_*10*_ = 18.385) but not in the task-irrelevant (Direction) condition (*t*_(23)_ = 0.239, *p* = 1, *BF*_*10*_ = 0.22).

Additionally, to assess the decisional carry-over effect, we grouped trials based on the preceding reports (“Short” or “Long” responses). Figure 4C displays psychometric curves for each group, revealing a distinct difference based on prior responses. As indicated in Fig. 4D, the PSE values for the prior “Long Response” was 934 ± 45 ms, significantly shorter than the prior “Short Response” (1166 ± 55 ms), revealing a significant decisional carry-over effect (*t*_*(23)*_ = 3.457, *p* = .002, *d* = 0.939).

### Omnibus analysis

**Fig. 5:**
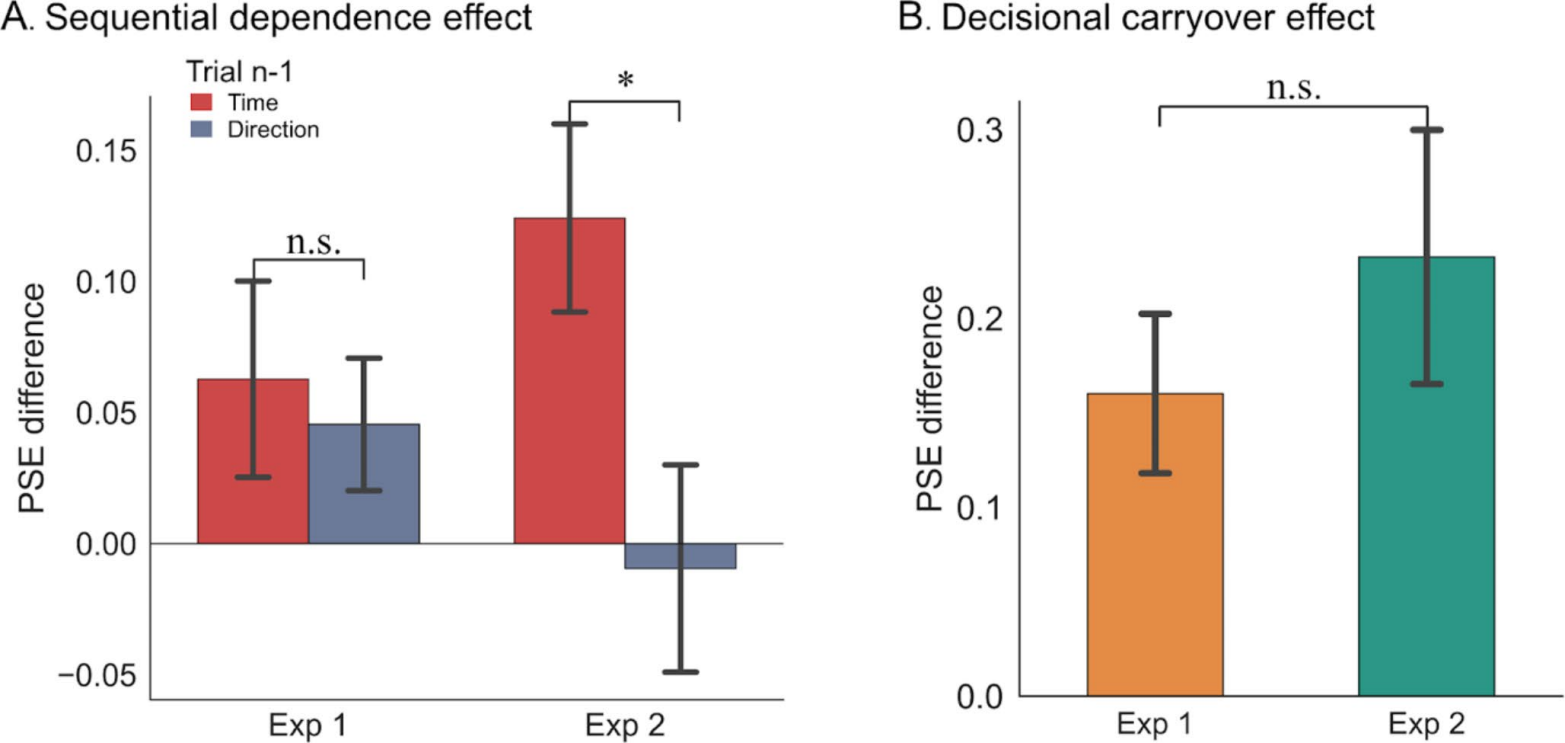
(**A**) The sequential dependence effects, measured by the difference of PSEs between Prior Short and Prior Long durations, are plotted separately for the preceding time-reporting (red) and direction-reporting (blue) trials, and Experiments 1 and 2. (**B**) Decisional carryover effects, measured by the difference of PSEs between Prior Short and Prior Long responses, are plotted separately for Experiments 1 and 2. Error bars represent ± SEM. *p < .05, while n.s. denotes non-significant

Our study aims to investigate the influence of task relevance on time perception in both time reproduction and time discrimination tasks. In Experiment 1, the preceding task-relevant response was the binary judgment (“shorter” or “longer”) in the discrimination task, while the preceding task-relevant response was the duration reproduction task in Experiment 2. Given that the task-relevance in two experiments was qualitatively different, we further conducted a nested ANOVA analysis to compare the sequential effects between the timing discrimination (Experiment 1) and the time reproduction (Experiment 2) tasks. The sequential effect index was calculated as the difference in PSEs between prior short and prior long durations for each prior task condition and for each experiment. A nested ANOVA on the sequential effect index, considering factors of the between-subject factor “Experiment” and the nested within-subject factor “Task Relevance”, revealed a significant interaction effect (*F*_(2,92)_ = 3.716, *p* = .028). However, there was no significant main effect of Experiment (*F*_(1,92)_ = 0.009, *p* = .927). Further paired *t*-tests on the PSE shifts for the difference of sequential effect between task relevance (Time vs. Direction) failed to reveal any significant difference in Experiment 1 (*t*_*(23)*_ = 0.371, *p* = .714, *d* = 0.110), but a significant difference in Experiment 2 (*t*_*(23)*_ = 2.341, *p* = .028, *d* = 0.723), indicating a more pronounced sequential dependence in trials with consecutive time reproduction tasks in Experiment 2 (Fig. 5A). The decisional carry-over effect index was calculated as the difference in PSEs between prior short and prior long reports separately for each experiment. A separate *t*-test on the decisional carry-over effect index did not show a significant difference between Experiments 1 and 2 (*t*_*(46)*_ = 0.911, *p* = .367, *d* = 0.263, see Fig. 5B).

Overall, both time discrimination and reproduction tasks demonstrated consistent assimilation toward prior durations, indicating a robust sequential effect in time perception. The comparative analysis revealed that task relevance enhanced sequential biases in the time reproduction task, but not in the time discrimination task. This suggests that the measurement type modulates the mechanism of sequential effect. The difference might stem from the interaction of the timing task with memory retrieval of the encoded duration, with the time reproduction task requiring continuous attention and memory comparison during reproduction.

## General discussion

The present study explored the impact of task relevance on sequential effects in time perception, using discrimination and reproduction tasks (Fornaciai et al., 2023; Togoli et al., 2021; Wehrman et al., 2023; Wiener et al., 2014). Across both timing tasks, we observed a consistent assimilation effect: participants perceived current durations as longer following long previous stimuli and shorter following short ones. Interestingly, while the assimilation effect with the discrimination task was unaffected by task relevance, it was more pronounced with the time reproduction task following the same task, highlighting distinct impacts of timing tasks on sequential dependence. Furthermore, we observed significant decisional carry-over effects in both timing tasks, where participants were more likely to repeat their responses, regardless of which timing task being used.

Our results indicated a significant sequential dependence effect in both duration discrimination and reproduction tasks, in line with previous findings in time perception (Glasauer & Shi, 2022; Togoli et al., 2021; Wehrman et al., 2023; Wiener et al., 2014). Recent past time intervals, being more accessible in memory, can influence the perception of current durations. In fact, recent studies argue that by integrating noisy sensory inputs with recent past stimuli (sequential effect) could enhance processing efficiency (Cheng et al., 2023; Fornaciai et al., 2023; Tonoyan et al., 2022), perceptual stability and temporal continuity (Cicchini et al., 2017; Fornaciai & Park, 2018a; Glasauer & Shi, 2022; Liberman et al., 2016). However, this also engenders byproducts, such as the central tendency and sequential biases. In this aspect, mechanisms of sequential dependence in time domain are comparable to those measured in non-temporal domains (Barbosa & Compte, 2020; Cicchini et al., 2014; Fischer & Whitney, 2014; Fornaciai & Park, 2018b; Kristensen et al., 2021; Manassi et al., 2018; Suárez-Pinilla et al., 2018; Turbett et al., 2021).

Interestingly, though, we found the influence of task relevance in the preceding trial on the current estimate showed distinctive patterns with different types of timing tasks. The task-relevant timing task displayed similar sequential effects to the task-irrelevant direction adjustment tasks, while the impact of the preceding timing task on the current duration reproduction was more pronounced compared with the preceding direction task. One plausible explanation lies in the differential memory processes engaged in reproduction and discrimination tasks. In the reproduction task, participants had to reactivate the encoded duration in working memory through the reproduction phase, as it was used as a reference for stopping the reproduction. This active maintenance was missing for the direction adjustment trials, leading to unequal sequential effects between reproduction-reproduction and direction-reproduction trials. The active memory trace of the target duration through the reproduction phase may thus bias the encoding of the subsequent trial. In contrast, the temporal bisection decision could be already made during the encoding phase, as it only requires the comparison of the target duration to the middle reference duration (here 1 s). Therefore, not much reactivation and memory processes are needed after the cue was presented, leading to comparable sequential effects between the preceding timing and non-timing tasks, as the decision could already be made prior the task cue. The enhanced sequential effect with consecutive reproduction tasks observed in the present study is inline with a recent fMRI study (Cheng et al., 2023), which also showed that consecutive responses enhanced sequential dependence. Their fMRI results revealed that sequential dependence negatively correlated with hippocampal activity in these consecutive response trials (Cheng et al., 2023), highlighting the crucial role of memory in sequential dependence (Bliss et al., 2017; de Azevedo Neto & Bartels, 2021).

Early decision criterion-setting accounts (see also Pascucci et al., 2023; Treisman & Williams, 1984) argued that the sequential effect depends on two opposing updating processes involved in setting decision criteria: the tracking and stabilization processes. The tracking process involves tracking recent sensory inputs, which biases decisions toward previous judgments, while the stabilization process reverts decision to a mean criterion set over a long-term process. An attractive sequential effect evolves when the tracking process is dominant. In our Experiment 2, the reproduction task requires more attention in monitoring the passage of time compared to the direction task, which likely strengthens the tracking process rather than the mean-reverted stabilization process for the consecutive reproduction trials. This boosted “internal attention” to the representation of a recently seen stimulus in working memory likely leads to an enhanced sequential effect.

However, this decision criterion-setting account, while explaining the influence of the task type on sequential effects, falls short when attempting to explain the comparable central tendency effects we observed. Recent work with an iterative Bayesian updating model (Glasauer & Shi, 2022) suggests that the short-term sequential effects are influenced by individuals’ beliefs in temporal continuity, whereas the long-term central tendency effect relies more on acquired sample distributions. The duration reproduction in our study, which requires ongoing monitoring, likely places more weight on temporal continuity compared to the temporal bisection task. This interpretation also helps to clarify why we observed an enhanced sequential effect in consecutive reproduction trials.

While we found distinct impacts of timing tasks on sequential dependence, strikingly, the decisional carryover effect, when the reproduction response was converted to binary category responses, was comparable between two timing tasks (see Fig. 4E). The decisional carryover effect we observed aligns with previous findings of response assimilation in duration judgments (Brown et al., 2005; Li et al., 2023; Wehrman et al., 2018, 2020, 2023; Wiener et al., 2014), particularly under conditions of response uncertainty (Akaishi et al., 2014; Wiener et al., 2014). Wehrman et al. (2023) suggest two potential possible explanations for this response assimilation: One is that response assimilation might actually reflect stimulus assimilation based on subjective rather than objective durations. When participants categorize a prior duration as “Short” or “Long”, they anchor their judgments of the subsequent stimulus accordingly, leading to judgments being assimilated to previous decisions (Urai et al., 2019; Wehrman et al., 2023). The second possibility involves the internal pacemaker, described in the classic internal clock model (Gibbon et al., 1984; Wearden, 1991), and assumes that the pacemaker’s rate fluctuates slowly and ‘sticks’ across multiple trials. This consistency, or ‘stickiness’, could give rise to response assimilation, as trials categorized based on preceding response outcomes (“Short” or “Long”) are likely in the same state of pacemaker rate as the preceding trial. Consequently, response assimilation is primarily driven by the ‘stickiness’ of the fluctuating pacemaker rate, rather than the task type or memory reactivation. While the anchoring account emphasizes that current decision-making is assimilated to an internal reference, the ‘sticky’ pacemaker account offers a mechanistic interpretation that is not limited to the late post-perceptual stage.

Both the anchoring account and the ‘sticky’ pacemaker account align with the concept of decisional inertia, proposed for non-temporal serial dependence (Ceylan et al., 2021; Pascucci et al., 2019), although decisional inertia emphasizes serial dependence occurring at the post-perceptual stage. Given that changes in decision states might rather be slow, decisional inertia exerts a stronger influence on decision judgments than the bias from the stimuli. Previous studies have also shown that the impact of decisional inertia can extend across different objects sharing the same decision, such as the orientation task (Ceylan et al., 2021; Fornaciai & Park, 2019; Huffman et al., 2018; Tanrikulu et al., 2023). However, decisional bias seems to operate independently of visual working memory (Pascucci et al., 2019), as also evidenced by the decisional carryover effect observed in the present study (see Fig. 5B). In this context, although decisional inertia can explain the decisional carryover effect, but the task relevance effect observed here may be more related to memory reactivation.

In conclusion, our findings highlight distinct impacts of timing tasks on sequential effects but reveal comparable patterns of response assimilation across tasks. While the temporal bisection task showed no changes in sequential effect by preceding task relevance, it was notably stronger in the duration reproduction task when it followed the same reproduction task, compared to a timing-irrelevant direction task. This enhanced sequential effect in consecutive reproduction tasks is likely owing to boosted attention and memory reactivation during the reproduction, absent in both the direction and the temporal bisection tasks. We also found comparable response assimilation across different timing tasks, which can be attributed to the influence of the pacemaker’s sticky rate and/or decisional inertia.

## Electronic supplementary material

Below is the link to the electronic supplementary material.

Supplementary Material 1

## Data Availability

The data and analysis code that support the findings of this study will be made available from the author, Si Cheng (chengsi123456@gmail.com), upon reasonable request. All data and code will be made available in online repositories upon acceptance.
