# Supplementary material for: The impact of task measurements on sequential dependence: a comparison between temporal reproduction and discrimination tasks
Source: Psychol Res. 2024 Aug 27;88(8):2346–59. doi: 10.1007/s00426-024-02023-x (PMC11522143; doi:10.1007/s00426-024-02023-x)
Supplement: Supplementary file 1 — Supplementary Material 1 [file 426_2024_2023_MOESM1_ESM.docx]

**Supplementary Materials**

**Analysis for direction reproduction trials**

Outliers due to accidental button presses or inattention were also excluded, specifically those with response errors larger than 45° for direction report trials, before proceeding with further analyses. These outliers were rare, constituting only 1.05% of direction report trials (ranging individually from 0 to 23 outlier trials) for Experiment 1 and 1.07% of direction report trials (ranging individually from 0 to 35 outlier trials) for Experiment 2. Next, we categorized the remaining trials into two categories based on the previous tasks: Time or Direction, to investigate the influence of prior tasks on the sequential effects of current direction estimates.

We focused on two conditions: the prior Direction task representing the prior task-related condition, and the prior Time task representing the prior task-unrelated condition. The direction of motion was randomly selected from a circular distribution, featuring 16 equally spaced angles (from 11.25° to 348.75°, in steps of 22.5°), which effectively neutralized any central tendency. Consequently, we focused solely on the sequential effect and skipped the central tendency analysis. The response error was calculated as the difference between the reported direction and the true motion direction for the current trial (i.e., estimate - direction). Negative errors indicated a counter-clockwise deviation from the true direction, while positive errors suggested a clockwise deviation. Additionally, the direction difference was also calculated between the current trial and the previous trial (the previous direction - the current direction), following the same method used in previous research [(e.g., Fischer & Whitney, 2014)](https://paperpile.com/c/rklA4N/0mBbU/?prefix=e.g.%2C%20). Trials with a direction difference of 0° or ± 180° were excluded, as response errors relative to these direction differences are undefined. Following previous research [(Moon et al., 2022)](https://paperpile.com/c/rklA4N/ENZuj) highlighting a significant role of non-directional orientation in the coding of visual motion direction, we reduced the direction difference range from [-180 to 180°] to [-90 to 90°] accordingly. To better reflect the repulsion and attractive biases, we converted the response errors from clockwise or counterclockwise directions to the repulsion (negative) and attractive (positive) biases by collapsing the direction differences to the positive range [0, 90.0°]. This analysis is akin to previous studies [(Bae & Luck, 2020)](https://paperpile.com/c/rklA4N/NykwC).

Prior research has shown that small orientation differences (within 90°) led to a significant attractive bias [(Bliss et al., 2017; Fritsche et al., 2017; Fritsche & de Lange, 2019; Samaha et al., 2019)](https://paperpile.com/c/rklA4N/i07xy+XEtOe+8975u+WBoU2). For example, attraction was observed when the orientation difference was around 17° [(Fritsche et al., 2017)](https://paperpile.com/c/rklA4N/XEtOe). Thus, we calculated the average response errors for small-orientation-difference trials (22.5°, 45.0° and 67.5°) and compared them to zero for each condition (the prior task being direction reproduction or duration reproduction for the current direction reproduction trials). The statistical significance of the sequential bias was assessed individually using two-sided *t*-tests against a null hypothesis of zero effect, and paired *t*-tests were run for within-subject between-condition comparisons.

**Results**

**Direction Estimation in Experiment 1.** The mean response errors were plotted against the orientation difference between the previous and the current trials (ranging from -90° to 90°, a positive value representing the difference in the clockwise direction), separated for the prior Direction and Time conditions (Figure S1.A). Then, the direction errors were converted to the attractive (positive) and repulsion (negative) sequential effect and replotted as a function of the absolute orientation difference for each condition (illustrated in Figure S1.B). The average response biases across the orientation differences of 22.5°, 45.0°, and 67.5° were 0.616° ± 0.399° and -0.040° ± 0.311° for the prior Direction and Time conditions. Both response biases did not significantly deviate from zero effect (prior Direction: *t_(23)_* = 1.541, *p* =.137, *d* =  0.315; prior Time: *t_(23)_* = -0.128, *p* =.899, *d* =  0.026) and there was no difference between the two, *t*_(23)_ = 1.588, *p* =.126, *d* =  0.374 (Figure S1.C). Experiment 1 didn’t reveal a significant attractive bias in the direction reproduction task.


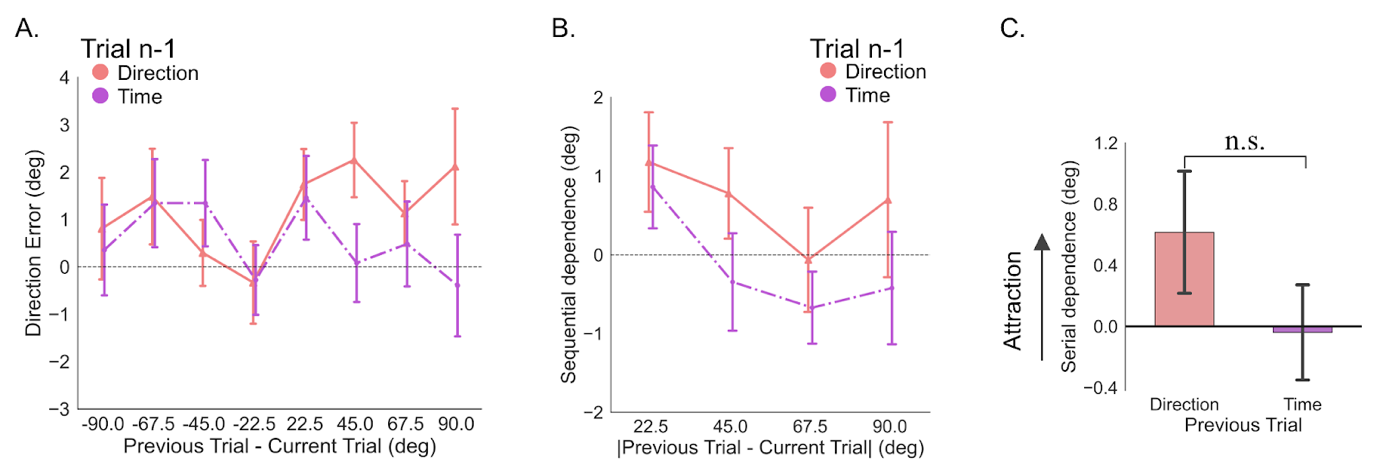
**Figure S1**. The results of direction reproduction trials for Experiment 1. (**A**) Mean response errors on the orientation difference of [-90°, 90°], plotted separately for trials preceded by Direction report and Time report. The angular difference was realigned to represent the relative motion orientation (plus 180° for the opposite direction) of the previous trial. (**B**) Mean errors on the absolute orientation difference of [0°, 90°], plotted separately for prior Direction and Time conditions. The sign of the response error was coded so that positive values indicate that the current-trial direction report was biased toward the direction of the previous trial, and negative values indicate that the current-trial direction report was biased away from the direction of the previous trial. (**C**) Mean errors averaged across 22.5°, 45.0°, and 67.5°, were plotted separately for prior Direction and Time conditions. Error bars represent ± SEM. n.s. denotes non-significant.

**Direction Estimation in Experiment 2.** Figure S2.A depicted the response errors against the orientation difference from -90° to 90° for prior direction reproduction and duration reproduction trials separately. The direction errors were translated into the attractive (positive) and repulsion (negative) sequential effect and replotted in Figure S2.B. The average response biases across the orientation differences of 22.5°, 45.0°, and 67.5° were 0.978° ± 0.294° and 0.469° ± 0.303° for the prior Direction and Time conditions. The averaged response biases were significant only for prior Direction task (prior Direction: *t_(23)_* = 3.333, *p* =.003, *d* =  0.680; prior Time: *t_(23)_* = 1.546, *p* = .136, *d* =  0.315) and there was no difference between the two, *t*_(23)_ = 1.167, *p* =.255, *d* =  0.348 (Figure S2.C).

To gain a better understanding of the differences between the two experiments, we further conducted an omnibus analysis across both experiments. A two-way mixed ANOVA on the attraction effect (averaged across 22.5°, 45.0°, and 67.5°) yielded neither a significant main effect for Experiment (*F*_(1,46)_ = 1.495, *p* = .228, p2 = 0.031), nor for the task relevance (*F*_(1,46)_ = 3.758, *p* = .059, p2 = 0.076). Additionally, there was no significant interaction effect between the two factors (*F*_(1,46)_ = 0.059, *p* = .809, p2 = 0.001).


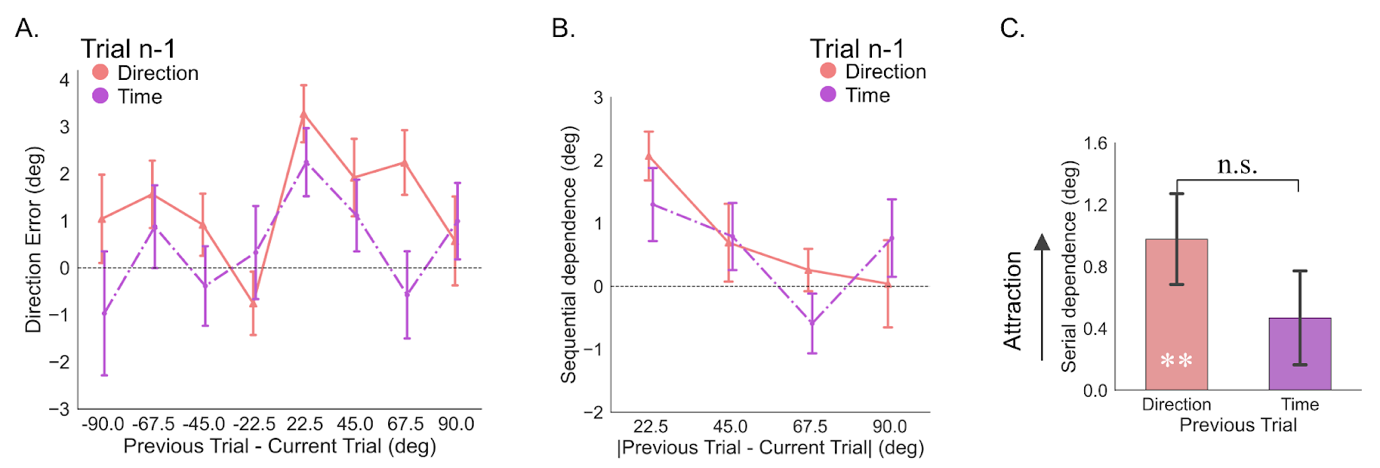


**Figure S2**. The results of direction reproduction trials for Experiment 2. (**A**) Mean response errors on the orientation difference of [-90°, 90°], plotted separately for trials preceded by Direction report and Time report. The angular difference was realigned to represent the relative motion orientation (plus 180° for the opposite direction) of the previous trial. (**B**) Mean errors on the absolute orientation difference of [0°, 90°], plotted separately for prior Direction and Time conditions. The sign of the response error was coded so that positive values indicate that the current-trial direction report was biased toward the direction of the previous trial, and negative values indicate that the current-trial direction report was biased away from the direction of the previous trial. (**C**) Mean errors averaged across 22.5°, 45.0°, and 67.5°, were plotted separately for prior Direction and Time conditions. Error bars represent ± SEM. n.s. denotes non-significant. ** *p* < .01, and n.s. non-significant.

The results in direction adjustment tasks showed that response biases in Experiment 1 for both preceding direction and time tasks did not significantly deviate from zero, indicating no significant sequential bias in the direction estimation task. However, in Experiment 2, significant attractive biases were observed only for the prior direction task. Considering both Experiments 1 and 2 adopted the adjustment tasks for direction trials, these distinct patterns of response biases in direction estimation tasks across different experiments may be due to the differential memory processes engaged in the encoding phase. For the duration reproduction, participants had to maintain the duration in working memory in the whole encoding phase to process it accurately, while the temporal bisection decision could be made before the end of the encoding display (the decision of “longer” was made after the encoding display passed 1 s, and no need to encode the whole display of 1.8 s). The omnibus analysis across both experiments revealed a marginally significant main effect for task-relevance (*F*_(1,46)_ = 3.758, *p* = .059, p2 = 0.076), suggesting that the attractive biases in direction estimates may also be task-relevant. However, there were no significant main effects for Experiment, nor a significant interaction effect between the two factors.

Although previous research has shown strong sequential effects in motion direction estimation, such as attraction [(Alais et al., 2017; Fischer et al., 2020; Moon & Kwon, 2022)](https://paperpile.com/c/rklA4N/LsJXc+3AGAs+U1zHT) or repulsion [(Bae & Luck, 2017, 2020; Kang & Choi, 2015)](https://paperpile.com/c/rklA4N/rvveV+htgjN+NykwC), the dominance of either effect remain mixed. Recent studies also suggest that attractive and repulsive biases can occur concurrently in motion direction processing [(Feigin et al., 2021; Fritsche et al., 2017; Moon & Kwon, 2022; Pascucci et al., 2019; Pascucci & Plomp, 2021; Sadil et al., 2024; Sheehan & Serences, 2023; Zhou et al., 2024)](https://paperpile.com/c/rklA4N/LsJXc+zsUuH+YCaIx+XEtOe+zLmlF+DTqCE+isi9M+WQLaX). For instance, a recent study showed that the preceding direction response induced an attractive bias, while the preceding motion direction caused a repulsion bias, both contributing to serial dependence  [(Moon & Kwon, 2022)](https://paperpile.com/c/rklA4N/LsJXc). In our study, we used coherent motion with relatively long exposure times (600 to 1800 ms). The adjustment task for determining motion direction, which required using the left and right arrow keys to modify the direction pointer, might induce significant decisional inertia from the previous response, leading to mixed attraction effects observed in our study.

**References**

[Alais, D., Leung, J., & Van der Burg, E. (2017). Linear Summation of Repulsive and Attractive Serial Dependencies: Orientation and Motion Dependencies Sum in Motion Perception. *The Journal of Neuroscience: The Official Journal of the Society for Neuroscience*, *37*(16), 4381–4390.](http://paperpile.com/b/rklA4N/U1zHT)

[Bae, G.-Y., & Luck, S. J. (2017). Interactions between visual working memory representations. *Attention, Perception & Psychophysics*, *79*(8), 2376–2395.](http://paperpile.com/b/rklA4N/htgjN)

[Bae, G.-Y., & Luck, S. J. (2020). Serial dependence in vision: Merely encoding the previous-trial target is not enough. *Psychonomic Bulletin & Review*, *27*(2), 293–300.](http://paperpile.com/b/rklA4N/NykwC)

[Bliss, D. P., Sun, J. J., & D’Esposito, M. (2017). Serial dependence is absent at the time of perception but increases in visual working memory. *Scientific Reports*, *7*(1), 1–13.](http://paperpile.com/b/rklA4N/8975u)

[Feigin, H., Baror, S., Bar, M., & Zaidel, A. (2021). Perceptual decisions are biased toward relevant prior choices. *Scientific Reports*, *11*(1), 648.](http://paperpile.com/b/rklA4N/zLmlF)

[Fischer, Czoschke, S., Peters, B., Rahm, B., Kaiser, J., & Bledowski, C. (2020). Context information supports serial dependence of multiple visual objects across memory episodes. *Nature Communications*, *11*(1), 1932.](http://paperpile.com/b/rklA4N/3AGAs)

[Fischer, & Whitney, D. (2014). Serial dependence in visual perception. *Nature Neuroscience*, *17*(5), 738–743.](http://paperpile.com/b/rklA4N/0mBbU)

[Fritsche, M., & de Lange, F. P. (2019). The role of feature-based attention in visual serial dependence. *Journal of Vision*, *19*(13), 21.](http://paperpile.com/b/rklA4N/i07xy)

[Fritsche, M., Mostert, P., & de Lange, F. P. (2017). Opposite Effects of Recent History on Perception and Decision. *Current Biology: CB*, *27*(4), 590–595.](http://paperpile.com/b/rklA4N/XEtOe)

[Kang, M.-S., & Choi, J. (2015). Retrieval-Induced Inhibition in Short-Term Memory. *Psychological Science*, *26*(7), 1014–1025.](http://paperpile.com/b/rklA4N/rvveV)

[Moon, J., & Kwon, O.-S. (2022). Attractive and repulsive effects of sensory history concurrently shape visual perception. *BMC Biology*, *20*(1), 247.](http://paperpile.com/b/rklA4N/LsJXc)

[Moon, J., Tadin, D., & Kwon, O.-S. (2022). A key role of orientation in the coding of visual motion direction. *Psychonomic Bulletin & Review*. https://doi.org/](http://paperpile.com/b/rklA4N/ENZuj)[10.3758/s13423-022-02181-2](http://dx.doi.org/10.3758/s13423-022-02181-2)

[Pascucci, D., Mancuso, G., Santandrea, E., Della Libera, C., Plomp, G., & Chelazzi, L. (2019). Laws of concatenated perception: Vision goes for novelty, decisions for perseverance. *PLoS Biology*, *17*(3), e3000144.](http://paperpile.com/b/rklA4N/DTqCE)

[Pascucci, D., & Plomp, G. (2021). Serial dependence and representational momentum in single-trial perceptual decisions. *Scientific Reports*, *11*(1), 9910.](http://paperpile.com/b/rklA4N/isi9M)

[Sadil, P., Cowell, R. A., & Huber, D. E. (2024). The push-pull of serial dependence effects: Attraction to the prior response and repulsion from the prior stimulus. *Psychonomic Bulletin & Review*, *31*(1), 259–273.](http://paperpile.com/b/rklA4N/zsUuH)

[Samaha, J., Switzky, M., & Postle, B. R. (2019). Confidence boosts serial dependence in orientation estimation. *Journal of Vision*, *19*(4), 25. https://osf.io/6uczk/.](http://paperpile.com/b/rklA4N/WBoU2)

[Sheehan, T. C., & Serences, J. T. (2023). Distinguishing response from stimulus driven history biases. In *bioRxiv* (p. 2023.01.11.523637). https://doi.org/](http://paperpile.com/b/rklA4N/WQLaX)[10.1101/2023.01.11.523637](http://dx.doi.org/10.1101/2023.01.11.523637)

[Zhou, L., Liu, Y., Jiang, Y., Wang, W., Xu, P., & Zhou, K. (2024). The distinct development of stimulus and response serial dependence. *Psychonomic Bulletin & Review*. https://doi.org/](http://paperpile.com/b/rklA4N/YCaIx)[10.3758/s13423-024-02474-8](http://dx.doi.org/10.3758/s13423-024-02474-8)
